# Integrated Processes Turning Pepper Sauce Waste into Valuable By-Products

**DOI:** 10.3390/foods12010067

**Published:** 2022-12-23

**Authors:** Jicheng Shu, Yongqin Yin, Zhijun Liu

**Affiliations:** 1 School of Renewable Natural Resources, Louisiana State University Agricultural Center, Baton Rouge, LA 70803, USA; 2 Key Laboratory of Modern Preparation of TCM, Jiangxi University of Traditional Chinese Medicine, Ministry of Education, Nanchang 330004, China; 3 College of Traditional Chinese Medicine, Guangdong Pharmaceutical University, Guangzhou 510006, China

**Keywords:** food waste, pepper sauce waste, by-products

## Abstract

Background: Safe and efficient disposal of millions of tons of pepper sauce waste (PSW) can be a challenge to pepper sauce manufacturers that are concerned about creating environmental hazards from the processing (e.g., preservative salt and vinegar) and intrinsic (e.g., the pungent capsaicins) ingredients. It will be immensely beneficial to process these waste materials before they go to disposal. This work presents integrated approaches for a complete utilization of waste materials by removing and recovering valuable by-products and/or ingredients while succeeding to minimal to zero hazards. Methods: Laboratory- and pilot-scale extraction processes were used to demonstrate the recovery of intrinsic compounds from PSW to pungent pepper oil. Flash chromatography was then applied to isolate the pungent capsaicins out of the pepper oil, thus generating a no-heat pepper oil. Results: By processing the waste materials, a number of valuable by-products with various yield percentages were produced. They included (1) hot red pepper oil (RPO, 8.0% *v*/*w*), (2) no-heat pepper residue (NHPR, 25.3% *w*/*w*), (3) no-heat red pepper oil (NHRPO, 4.0% *v*/*w*), (4) capsaicinoids (0.8% *w*/*w*), and (5) capsaicin (0.4% *w*/*w*). The optimum processing conditions for products 1, 2, and 3 include extracting the waste materials with 95% ethanol twice, each lasting three hours. The optimal isolation conditions of flash column chromatography to obtain products 4 and 5 include the use of the pre-packed chromatography column 130 g Redisep C18, preparing the sample concentration to 100 mg/mL, eluting with aqueous ethanol, and detecting capsaicins at a wavelength of 228 nm. Conclusions: An integrated approach is offered for the complete utilization of PSW. It not only turns organic food waste into numerous new commodities but also significantly reduces the volume and degree of potential environmental hazard to the disposal sites.

## 1. Introduction

Pepper sauces, also known as chili sauces or hot sauces such as the renowned Tabasco™ sauce (McIlhenny Co., Avery Island, LA, USA) enable diners to enhance meal flavor. They are a popular addition to many different types of cuisines in countries like China, United States, Japan, India, and many more. Presently the world produces around 8.0 million tons of pepper sauces according to the report of Zero Power Intelligence Industry Research Center [1]. Because pepper sauces are primarily pepper flesh, the rest of the structural and fibrous materials are generated during the production process. These leftover materials include the lump of fibrous connecting tissues, peels, and seeds, along with processing ingredients such as salt, vinegar, and water. While the pepper sauces continue onto the marketplace, the leftover materials often become waste that must be disposed. Disposing these materials becomes a dilemma because of the huge volume and the remaining ingredients in them. Among the ingredients are salt, vinegar, oleoresin, Capsicum red or capsanthin (carotenoids pigments), and pungent capsaicinoids. Capsaicinoids is a homologous series with capsaicin and dihydrocapsaicin being the most abundant structures. The output of pepper sauces brings about enormous amounts of pepper sauce waste (PSW) and along with the preservative chemicals presents an unbearable environment pressure at disposal sites. It is clearly becoming a serious operational problem for these manufacturers. On the other hand, some of the remaining plant ingredients are food, spice, pesticidal, and medicinal, presenting incentive and opportunities to create additional value-added products to the industry [2].

The main issue has to do with the pungent capsaicin and its natural analogues, collectively referred as capsaicinoids. Capsaicin is responsible for the food flavor. It is also a pharmaceutical ingredient in the over-the-counter pain relief cream, e.g., 0.1% capsaicin cream and prescription drug [3], e.g., orthopedic pain relief patch Qutenza^®^ containing 8% capsaicin [4]. Moreover, capsaicin is used as the active ingredient in pesticides to deter animal and herbivory attacks. Bear spray, for example, contains 1.3% capsaicin [5]. Pepper spray maze in personal defense against crimes contains approximately 10% capsaicin [6]. Obviously, capsaicin can induce strong biological responses by hitting the transient receptor potential vanilloid type 1 ion channel (TRPV1), also known as the capsaicin receptor [7]. Therefore, disposal and release to the environment can have a dramatic impact on wildlife, soil biology, and soil chemistry, among other aspects. Even though the hot pepper sauces took away some of the capsaicin, majority of it remains in the seeds of these leftover materials. Determination showed that some 80% of capsaicin was still in the waste materials with a content of approximately 1.0–3.5 mg/kg [8]. The general consensus is that if capsaicin is largely or completely removed from these materials before disposal, then the chemical and environmental impact will be minimized to safer levels. Clearly the key issue lies at the removal of capsaicin from the leftover materials.

It is perhaps the need for the removal of capsaicin that drives recovery. Recovering capsaicin before disposal opens a series of utility avenues. For example, in addition to food, pharmaceutical, pesticidal, and personal defense uses, capsaicin has been used industrially as the active ingredient in ship painting to ward off parasites in ocean and fresh waters [9]. This industrial use alone can absorb a sizable amount of recovered capsaicin thus turning the removal step into a desirable recovery process.

Removing capsaicin costs undoubtedly and can become cost-prohibitive in front of a low recovery efficiency. However, if an effective and scalable processing method can be developed, the cost of obtaining a marketable capsaicin and a series of value-added products from the waste materials may offset the cost of disposing it in an environmentally sound way. Unfortunately, few research and report focused on the PSW to the best of our knowledge. Most of the manufacturers would embrace the idea of recovery but are concerned about not having feasible and practical methods for achieving it. According to the current management protocols of food waste, obtaining by-products from the waste by recycling technology is an optimal method desired by most manufacturers. PSW is a storehouse of multiple natural compounds, among which are oleoresin, the non-pungent oleoresin-soluble red pigment capsanthin, and the pungent oleoresin-soluble capsaicinoids. The red pigment in the oleoresin is a favorite coloring and flavoring product for the food industry. Depending on the content of capsaicinoids, the pepper red oil can be none-pungent or pungent. In addition to its attribute to pungency taste, the alkaloidal capsaicinoids has many reported medicinal properties that include cardio protection, anti-lithogenic effect, anti-inflammation, analgesia, thermogenic influence, and beneficial effects on the gastrointestinal system [10]. With such valuable natural products potentially going to the waste and imposing environmental impact, it is well justified to invest in resources to reverse both fates. The present work reports our attempt to find an efficient and practical method for recovering capsaicinoids and the results of adopting integrated approaches for a complete utilization of pepper sauce waste. The feasibility for recovering valuable natural ingredients for developing new spin-off products before proceeding to cleaner and safer disposal is presented.

## 2. Materials and Methods

### 2.1. Experimental Materials

The PSW was graciously provided by the Department of Research and Development of McIlhenny Co. (Avery Island, LA, USA). The pepper sauce was made out of cayenne pepper (Capsicum annuum) grown in South America with confidential variety, specific location, harvesting and processing methods, and source desired by the manufacturer. The PSW was wet, containing approximately 67% aqueous liquid and 33% oily solid, based on an oven-drying preliminary study. The reference compounds of capsaicin and dihydrocapsaicin were obtained from Sigma-Aldrich Co. (St. Louis, MO, USA). All chemicals used in the laboratory were of analytical grade.

### 2.2. Extraction Methods

#### 2.2.1. Solvent Selection and Optimization of Extracting Condition

Among various solvents available, the ethanol and water binary system was selected. The extracting condition was investigated by mono-factor analysis. On the basis of production safety, the extracting temperature was set at room temperature. Three factors including the concentration of aqueous ethanol, extracting time, and extracting frequency, were examined in this experiment. A total of 9 experiments were carried out. Each experiment was performed in triplicate. Both the content of capsaicinoids determined by HPLC and the peppery taste were taken as responses. 

#### 2.2.2. Crude Extraction

Crude extraction refers to the first extraction of the PSW that separates extractable from unextractable materials. PSW materials, about 15 kg, were extracted with 75 L of 95% aqueous ethanol in an agitated plastic tank at room temperature for three times, each time lasting 5 h. The ethanolic extract was allowed to sit still for 2 h to reach gravity separation of solvent from solid. The supernatant was then pumped out (Masterflex L/S, Cole-Parmer Instrument Co., Vernon Hills, IL, USA). The lower layer was centrifuged at 4000 rpm/min for 10 min in an Allegra 6KR centrifuge (Beckman Coulter, Indianapolis, IN, USA) to recover the remaining ethanolic extract. The ethanolic extracts were combined and named E-1. The remaining unextractable solid residue was named SR-1.

#### 2.2.3. Purification of Crude Extract

A 20-L rotary evaporator (R-220, Buchi Rotavapor®, Switzerland) was used to concentrate the ethanolic extract obtained above, yielding a crude extract referred as the crude red pepper oil (RPO). The crude RPO was a mixture of oil/oil-soluble compounds and water/water-soluble compounds. To separate this mixture, the crude RPO was washed with water three times. The process of washing was as follows: the crude red pepper oil was dispersed in 1.8 L of warm water (60 °C) in a 5-L glass beaker. The beaker was set in a thermostatic water bath (60 °C) for 30 min. A 5-L separatory funnel was used to separate the oil from the water. The HPLC analysis should be showed that little (below the limit of quantitation) capsaicinoids ended up in the water layer, which mainly contained the water-soluble salt and vinegar. The crude RPO was cooled to room temperature and centrifuged at 8000 rpm/min for 15 min (Centrifuge 5810R, Eppendorf, Germany) to remove insoluble impurities.

#### 2.2.4. Secondary Extraction of Solid Residue

Although the first ethanolic extraction took majority of the pungent ingredients out of the beginning material PSW and put them into the hot RPO product, the remaining oil-less pepper seeds in SR1 still contained a tiny amount (about 3.2 μg/kg) of capsaicinoids. Capsaicinoids are responsible for the pungency of pepper. In order to completely remove the pungency, the solid residue was subjected to a second round of an aqueous ethanolic extraction. This process was meant to result in a material (mostly seeds) that was no longer pungent shown by an undetectable level of capsaicinoids by HPLC. To achieve this, SR-1 was first crushed in a high-speed blender (Heavy Duty Blender, Waring Laboratory, USA), then extracted once with 95% aqueous ethanol in an extraction vessel. The vessel was placed on a heavy-duty orbital shaker at room temperature for 1 h and let sit to settle for 1 h. The supernatant was pumped out and named E-2. The lower layer was centrifuged at 4000 rpm/min for 10 min to remove the remaining liquid extract. After liquid extraction, the solid residue was named SR-2. SR-2 was washed with water three times. Briefly each time, SR-2 was dispersed in 10 L hot water (60 °C) in an agitated 20 L percolation bucket. After 30 min, water was discharged. The solid residue was dried at 105 °C for 2 h to yield the no-heat pepper residue (NHPR).

### 2.3. Isolation Methods for Capsaicinoids

#### 2.3.1. Chromatographic Fractionation of RPO

E-2 was concentrated by a rotary evaporator to yield the residue. The residue of E-2 and hot RPO were jointly dissolved in 95% ethanol to a concentration of 100 mg/mL. The mixture was subjected to the flash chromatography. The flash chromatography system was Combi Flash with multi-wavelength detection (Tyledyne Isco, NE, USA). The flash column was 130 g RediSep Reversed-phase C-18 column (Tyledyne Isco, NE, USA). The gradient of mobile phase was designed for separating capsaicinoids (Table 1). Ten milliliter of the above prepared solution was injected into the instrument for isolating. Fraction A (capsaicinoids) and fraction B (pepper oil) were collected. Fraction A was concentrated by a rotary evaporator until a white suspension spot appeared. The concentrate containing the white suspension spot was then poured into a beaker and covered with a glass lid. The beaker was kept in refrigerator (4 °C) for at least 24 h to allow the growth of crystals. Before filtering, chucky crystals were visible and the white suspension spot disappeared. The crystals were separated from the liquid by using a 0.45 µm membrane, air-dried and collected in a fume hood. Fraction B was concentrated until nearly all ethanol/water was evaporated (no more condensed solvent drips in the water-chilling condenser) and nearly all was oil. Lastly, the above oil was poured into a separatory funnel, added water, shaken lightly, and let stand and separate into oil (top)/water layers. The upper oil layer is the product referred as the no-heat RPO (NHRPO), a fraction of the RPO without the pungent compounds.

#### 2.3.2. Isolation of Capsaicin from Capsaicinoids

**Figure 1:**
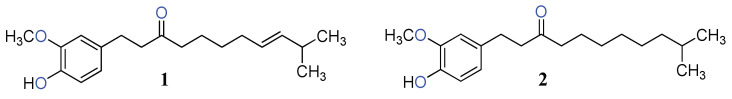
The structure of capsaicin (**1**) and dihydrocapsaicin (**2**).

Capsaicin and dihydrocapsaicin (Figure 1) are two major capsaicinoids in hot pepper oil, which are responsible for about 90% of the pungency [11,12]. Therefore, the isolation of the two capsaicinoids from the rest with high purity (e.g., 95% pure) and yield has great industrial applications to support pharmacological research and clinical uses [13]. 

The capsaicinoids obtained from the fractionation step above were dissolved in 95% ethanol to a concentration of 100 mg/mL. The sample was subjected to the same flash chromatography as above. The gradient of mobile phase was designed for separating the single compound capsaicin (Table 2). Sample was injected into instrument for isolating. Fraction C (capsaicin) was collected. Fraction C was concentrated by evaporator. The concentrated solution was placed in a beaker and kept in a refrigerator (4 °C) for at least 24 h to allow the growth of crystals. The crystals were filtered through a 0.45 µm membrane and air-dried in a fume hood to yield capsaicin.

### 2.4. HPLC Analysis

HPLC analysis was performed on a Waters 600E system with an auto sampler and a photodiode array detector (Waters Com., Milford, MA, USA). The analysis was conducted on a Phenomenex Luna C18 column (250 mm × 4.6 mm, 5 µm). Mobile phase A consisted of HPLC grade acetonitrile; mobile phase B consisted of HPLC-grade water containing 0.2% *v/v* phosphoric acid. The gradient eluting mobile phase was schemed to be from 0 to 50 min, A/B (25:75, *v*/*v*) to A/B (75:25, *v*/*v*) and from 50 to 60 min, A/B (75:25, *v*/*v*) to A/B (100:0, *v*/*v*). Mobile phase was pumped at 1.0 mL/min, the column temperature was maintained at 30 °C, and the injection volume was 5.0 µL. The wavelengths of PDA detection ranged from 200 to 600 nm and capsaicinoids detected at 228 nm. Capsaicin and dihydrocapsaicin were identified by running the reference compounds under the same HPLC conditions and matching each’s retention time and UV absorption spectrum.

## 3. Results

### 3.1. Extracting Condition

The extracting condition was investigated by mono-factor analysis. The best condition for solvent extraction was 95% aqueous ethanol, 5 h per extraction, and three times (Table 3).

### 3.2. Product of RPO

The E-1 was combined to a total volume of about 200 L. In the meantime, 4.5 kg SR-1 was yielded. The end product, hot RPO of 1.2 L, was clear and smelled the aroma similar to a typical Tabasco™ sauce product. The physical properties of the hot RPO displayed typical color, flavor, and density seen in some of the commercial pepper oil products. The yield of hot RPO was about 8% v/w of the beginning waste material of 15 kg PSW.

### 3.3. Product of NHPR

**Figure 2:**
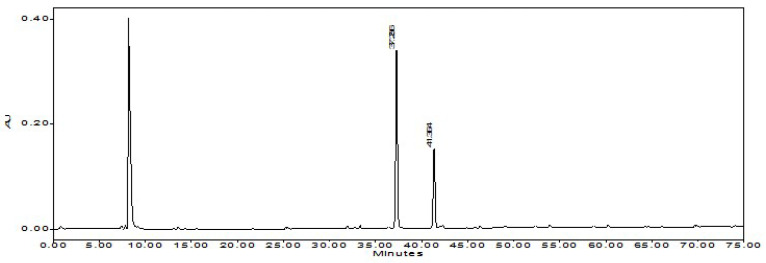
The chromatogram of reference compounds of capsaicin and dihydrocapsaicin.

**Figure 3:**
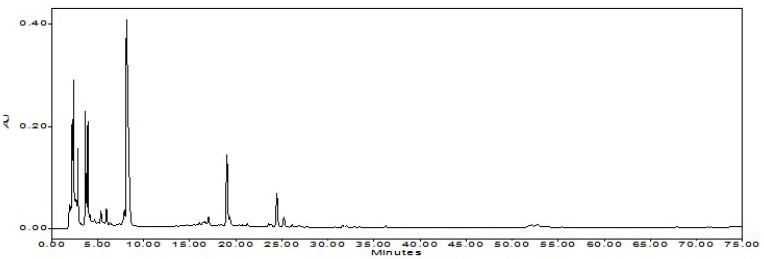
The chromatogram of NHPR.

After the second ethanolic extraction, 4.2 kg of solid residue (named SR-2) was yielded. SR-2 was further dried to yield 3.8 kg NHPR (25.3% *w*/*w* of the beginning waste materials PSW). The product was faint yellow, taste characteristic of Tabasco™ pepper sauce but not hot. Furthermore, HPLC analysis showed that the solid residue did not contain detectable capsaicinoids. The chromatograms of NHPR with reference compounds of capsaicin and dihydrocapsaicin were shown in Figure 2 and Figure 3. The retention time (Rt) of capsaicin was 37.3 min, and the Rt of dihydrocapsaicin was 41.3 min.

### 3.4. Product of Pure Capsaicinoids and NHRPO

**Figure 4:**
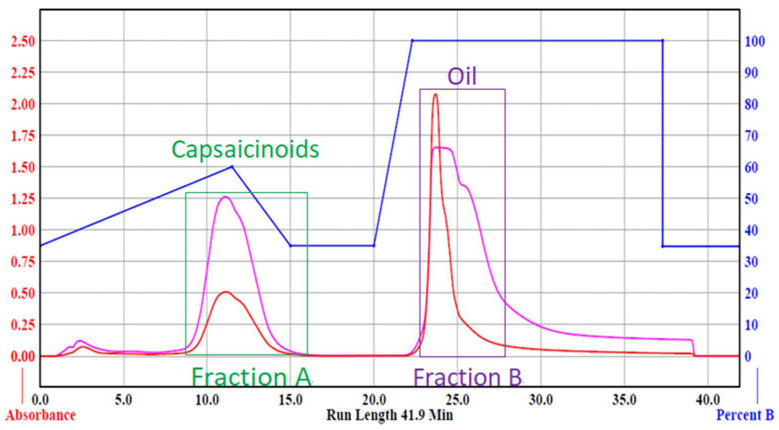
Chromatograms of capsaicinoids (Fraction A) and no-heat red pepper oil or NHRPO (Fraction B). The blue line indicates the percentage of ethanol in the aqueous mobile phase. The pink and red lines indicate the UV absorptions at 228 nm (for detection) and 300 nm (for monitoring), respectively.

The residue of E-2 and hot RPO were subjected to the flash chromatography. The elution from 9 min to 16 min of the chromatogram was fraction A (capsaicinoids), and the elution from 23 min to 28 min was fraction B (pepper oil) (Figure 4).

**Figure 5:**
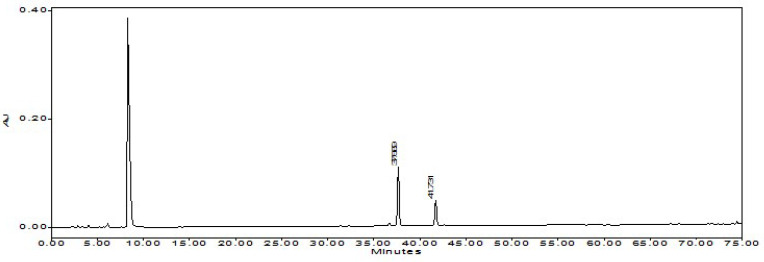
The chromatogram of capsaicinoids.

**Figure 6:**
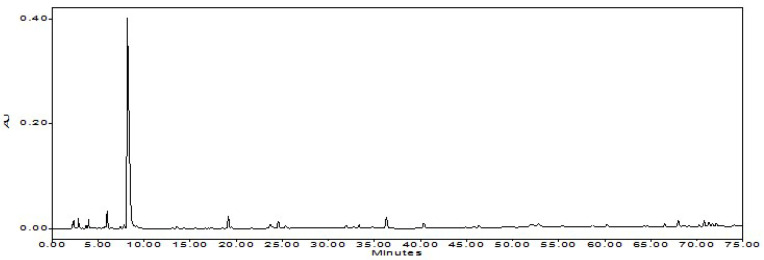
The chromatogram of NHRPO.

120 g capsaicinoids (primarily capsaicin and dihydrocapsaicin) and 0.6 L NHRPO were obtained from the combined E-2 and hot RPO (1.2 L) material. The yield of capsaicinoids and NHRPO were, respectively, 9.2% and 50%, which were equal to 0.8% *w*/*w* and 4.0 % *v*/*w* of the beginning waste material PSW. The capsaicinoids as a total capsaicinoids product has a purity of 98% as determined by HPLC. NHPRO contains non-detectable capsaicinoids, thus no longer pungent or hot (Figure 5 and Figure 6).

### 3.5. Product of Capsaicin

**Figure 7:**
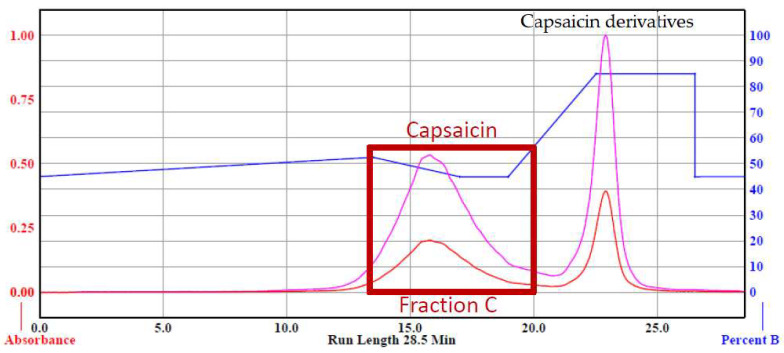
Chromatogram of capsaicins showing the separation of capsaicin (Fraction C) from other capsaicin derivatives. The blue line indicates the percentage of ethanol in the aqueous mobile phase. The pink and red lines indicate the UV absorptions at 228 nm (for detection) and 300 nm (for monitoring), respectively.

The capsaicinoids were injected into instrument for isolating. The elution from 14 min to 20 min was fraction C (Figure 7).

**Figure 8:**
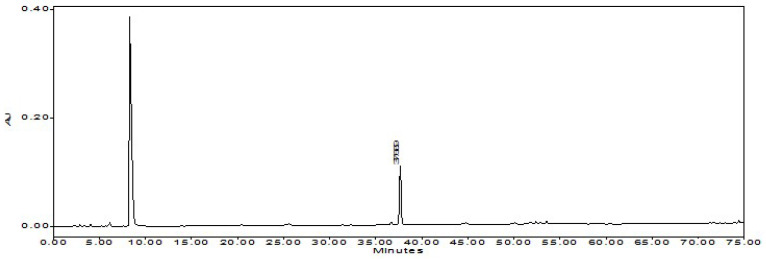
The chromatogram of isolated capsaicin.

When all capsaicinoids were processed, 60 g of pure capsaicin was obtained. The purity of isolated capsaicin was 95% based on HPLC analysis (Figure 8). The yield was 50% from the capsaicinoids or 0.4% *w*/*w* of the beginning waste material PSW. The unaccounted was assumingly lost during the process.

### 3.6. Spin-Off Products Produced during an Integrated Process of PSW

**Figure 9:**
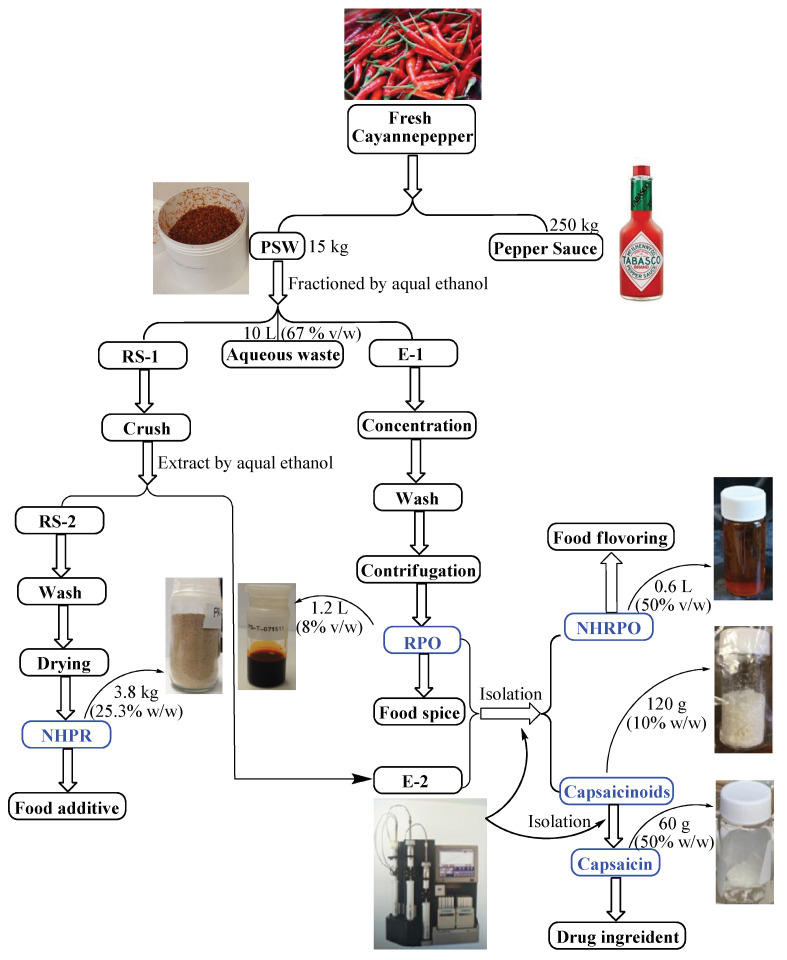
Overview of an integrated process for PSW utilization.

The beginning material was fresh cayenne pepper harvested from the field. It was subjected to a long process (approximately 2 years) by the action of salt and vinegar as processing and preserving ingredients in a sealed woody container. At the due time, the sauce was extracted and of course the rest went to waste. Roughly one ton of fresh pepper produces 250 kg sauce and 15 kg PSW, wet with salt and vinegar residues along with endogenous pepper natural ingredients. This study started with the PSW. The schematic integrated processes for a comprehensive PSW utilization were illustrated in Figure 9. As one can see, the 15 kg of PSW was transformed three fractions of approximately 3.8 kg of dry no-heat pepper residue (NHPR), 1.2 L of hot red pepper oil (RPO), and aqueous waste containing water-soluble ingredients of salt, vinegar, and others. This completed the first round of processing resulting in non-heat solid material and hot liquid as potential new products as well as liquid waste. The dry NHPR, despite of becoming non-pungent, still retains the same flavor of the sauce product after a second ethanolic extraction (i.e., Tabasco™) but mostly free of salt and vinegar. This material now can be used for creating new food products or food additives, for instances, to flavor cake, dessert, biscuit, and feed for animals such as pets, cow, and pigs. All pungent capsaicinoids and red pepper pigment were rounded into the hot RPO fraction. The second round of processing was thus devoted to separating the pungent capsaicinoids out of the hot RPO oil. After the capsaicinoids (10% by weight) were removed, the remaining oleoresin (50% by volume) was no longer hot but still red as the pigment remained, referred as no-heat RPO (NHRPO). The unaccounted portion got assumingly lost during the process. When further isolation was conducted, capsaicin was isolated to 99% purity by half from the other half other capsaicins. NHRPO can be used in a wide variety of applications as coloring agent, food, health, and pharmaceutical products. The bioactive compounds capsaicinoids can be used in the preparation of pharmaceutical or nutraceutical products, e.g., for cholesterol lowering and anti-cancer medicine, and other products such as defensive spray. In all processes, only ethanol and water were used. The pepper waste can be potentially transformed to five new products as described above. The volume for disposal was significantly reduced to as low as zero, other than the salt and vinegar that were cleaned of the waste materials.

## 4. Discussion

Recovering valuable ingredients from food and fruit waste has become increasingly attractive [14,15,16]. In order not to add hazardous waste from processing methods, green extraction techniques have been sought [17,18]. Three main extraction methods, namely the mechanical extraction, Supercritical CO_2_ fluid extraction (SFE) and solvent extraction, are readily available and widely used [19]. The mechanical extraction method is often associated with low yields and the starting subject for extraction should be dry-solid. However, the PSW contained liquid and was semi-solid. Before using this method, wet waste must be first dried into solid. This process was difficult and could be a costly step that manufacturers want to avoid. The SFE method has become one of the most promising food processing technologies. It offers numerous potential advantages over conventional extraction methods including being nontoxic, nonexplosive, and environmentally friendly [20,21]. However, this process is applicable for solid sample thus like the mechanical extraction method the material must be first dried. Being able to only process a small volume of material is another limitation [22], let alone the startup cost of expensive apparatus, setting up, and operation. The solvent extraction method in majority of the cases employs organic solvents such as hexane, which are less safe to handle and unacceptable to some manufacturers due to concerns over the harms they can cause to human health and the environment. For a possible industrial application, the choice of green-based natural sources, industrial-scale manufacturing, and safer solvents employed for the extraction process becomes essential for success. According to the above analysis, the selection of ethanol and water binary system for the extraction of PSW meets the criteria. Ethanol is recoverable and can be re-cycled for uses. The use of aqueous ethanol appears to be favored when it comes to food waste processing [23]. 

Because of similarity in the polarity and physical properties among capsaicinoid derivatives and non-capsaicinoid constituents, it was difficult to isolate by the simple and readily available industrial-scale manufacturing methods such as recrystallization and distillation. The chromatography techniques were the recognized methods for isolating capsaicinoid derivatives. However, the conventional column chromatographic approaches were not only costly, but also difficult for scale-up in the industry. Flash chromatography, also known as medium pressure chromatography, differs from the conventional technique in two ways: first, slightly smaller silica gel particles (250–400 mesh) are used; and second, due to restricted flow of solvent caused by the small gel particles, pressurized gas (ca. 10–15 psi) is used to drive the solvent through the column stationary phase. The net result is a rapid (“over in a flash”) and high-resolution chromatography [24,25]. Based on these assumptions, this column chromatography was predicted to be better for the isolation of capsaicinoids from RPO than the conventional open-top column chromatography.

Disposal of pepper sauce waste containing capsaicinoids is problematic to the environment and imposes a serious operational problem for the pepper sauce industry. In the meantime, capsaicin and its analogues are valuable commodity that is otherwise thrown away. In addition to the capsaicinoids, oleoresin oils, natural pigments, and flavored materials can also be utilized for creating new products. The current study examined undertaken processes that can lead to value added materials and products and concluded the feasibility of taking an integrated technical approach to alleviating both disposal concerns and adding commodity items. At least six value-added materials can be obtained as demonstrated in this study to create new products by processing the concerned pepper sauce waste. They include pungent crude extract, pungent red pepper oil, non-pungent and flavored biomass such as pepper seeds, non-pungent and flavored red pepper oil, pungent total capsaicinoids, and the single pungent compound capsaicin. All pungent materials can be used to create pepper spray for various defense functions, parasite-deterring paint for the shipping industry, pharmaceutical-grade medicines, and capsaicin-activated medical devices. The non-pungent and flavored ingredients can be used to develop new food spice and additive products. In the meantime, even if the non-pungent materials were of little interest to further the product pathway, the waste containing a minimal amount of capsaicinoids no longer imposes environmental and biological hazards.

Our study demonstrated the above feasibility. Using 15 kg PSW derived from 250 kg fresh pepper it was shown that new products can be yielded: (1) 1.2 L of hot red pepper oil (RPO) that can be used in flavoring. (2) 3.8 kg of dry no-heat pepper residue (NHPR) that can be developed into food flavoring and additives due to its retaining of the well-recognized sauce (e.g., Tabasco) flavor; (3) 0.6 L of no-heat but flavored red pepper oil (NHRPO) that can be used as a food coloring agent; (4) 120 g capsaicinoids that can be used for health care, personal defense, and organic pesticides; (5) 60 g pure capsaicin that can be used as a pharmaceutical-grade medicinal ingredient; and (6) the pungent crude extract right out of the pepper sauce waste if one does not want to go down the complete path of processing as described above for making functional food and supplements. Through further refining and defining, all the processes demonstrated by this study could be made green (i.e., no synthetic solvents or chemicals involved) including extraction and isolation. Thus, the above by-products recovered from the PSW added values and could offset the cost of operation and eliminate the disposal burden and expenses.

## 5. Conclusions

Pepper sauce waste was processed in an integrated process, creating a series of by-products that retain the original sauce flavor and/or pungency, or become the source of pharmaceutical-grade capsaicin. With the use of a green and recoverable solvent, the only waste generated from the otherwise pepper sauce waste can now be reduced to a liquid containing water-soluble vinegar, salt and others. Adding values and reducing waste are shown to be simultaneously achievable by using an integrated process reported in this study.

## Figures and Tables

**Table 1:** The Gradient of mobile phase for separating capsaicinoids.

Duration (min)	% B	Solvent A	Solvent B
0.0	35.0	Water	EtOH
11.5	60.0	Water	EtOH
3.5	35.0	Water	EtOH
5.0	35.0	Water	EtOH
2.5	100.0	Water	EtOH
17.5	100.0	Water	EtOH

**Table 2:** The gradient regimen of mobile phase for separating capsaicin and capsaicinoids.

Duration (min)	% B	Solvent A	Solvent B
0.0	45.0	Water	EtOH
13.5	52.5	Water	EtOH
3.5	44.9	Water	EtOH
2.0	44.9	Water	EtOH
3.6	85.0	Water	EtOH
4.0	85.0	Water	EtOH
2.0	45.0	Water	EtOH

**Table 3:** Extracting factors and results of PSW.

Run	Factor	Levels	Peppery Taste	Capsaicinoids
1	Concentration	100%	−	−
2		95%	−	−
3		90%	−	+
4	Time (h)	1	+	+
5		3	−	+
6		5	−	−
7	Frequency	1	+	+
8		2	+	+
9		3	−	−

“−” was negative result; “+” was positive result.

## Data Availability

Data is contained within the article.
